# Strain imaging in heart failure: from early detection to prognostic assessment

**DOI:** 10.1093/eschf/xvag232

**Published:** 2026-09-30

**Authors:** Wojciech Kosmala, Anna Węgrzyn-Witek

**Affiliations:** Faculty of Medicine, Institute of Heart Diseases, Division of Cardiovascular Imaging, Wroclaw Medical University, ul Borowska 213, Wroclaw 50-556, Poland; Institute of Heart Diseases, Division of Cardiovascular Imaging, Jan Mikulicz Radecki University Hospital, ul Borowska 213, Wroclaw 50-556, Poland; Institute of Heart Diseases, Division of Cardiovascular Imaging, Jan Mikulicz Radecki University Hospital, ul Borowska 213, Wroclaw 50-556, Poland

**Keywords:** Heart failure, Strain imaging, Speckle tracking echocardiography, Early detection, Prognosis

## Abstract

Traditional left ventricle ejection fraction (LVEF)-centric approaches often detect cardiac dysfunction late and offer limited insights into myocardial mechanics. Myocardial strain imaging by two-dimensional speckle-tracking echocardiography has emerged as a high-sensitivity alternative for comprehensive evaluation of heart failure (HF). This synthesis evaluates the diagnostic, prognostic, and therapeutic monitoring capabilities of multi-chamber strain imaging across the HF spectrum. Based on current evidence, global longitudinal strain (GLS) provides superior sensitivity over LVEF in detecting subclinical myocardial injury. In preclinical settings (Stage B HF), a GLS <16% is an important diagnostic criterion, reclassifying 13% of asymptomatic patients and predicting a four-fold higher risk of HF hospitalization. In cardio-oncology, a 15% relative decline in GLS successfully identifies subclinical toxicity before LVEF drops. Across HF phenotypes, GLS <16% has been incorporated as a minor diagnostic criterion for heart failure with preserved ejection fraction (HFpEF). GLS is a 1.6-fold better prognostic marker than LVEF (particularly in preclinical stages and HFpEF) and exhibits a more linear relationship with mortality. Furthermore, left atrial reservoir strain (<18%) accurately grades diastolic dysfunction, while right ventricular free-wall longitudinal strain acts as a powerful independent predictor of death. In conclusion, strain imaging has expanded from a specialized technique to a part of contemporary HF evaluation. By capturing myocardial mechanics, it enables early detection, precise phenotyping, robust risk stratification, and timely therapy monitoring.

## Graphical Abstract

**Graphical Abstract:**
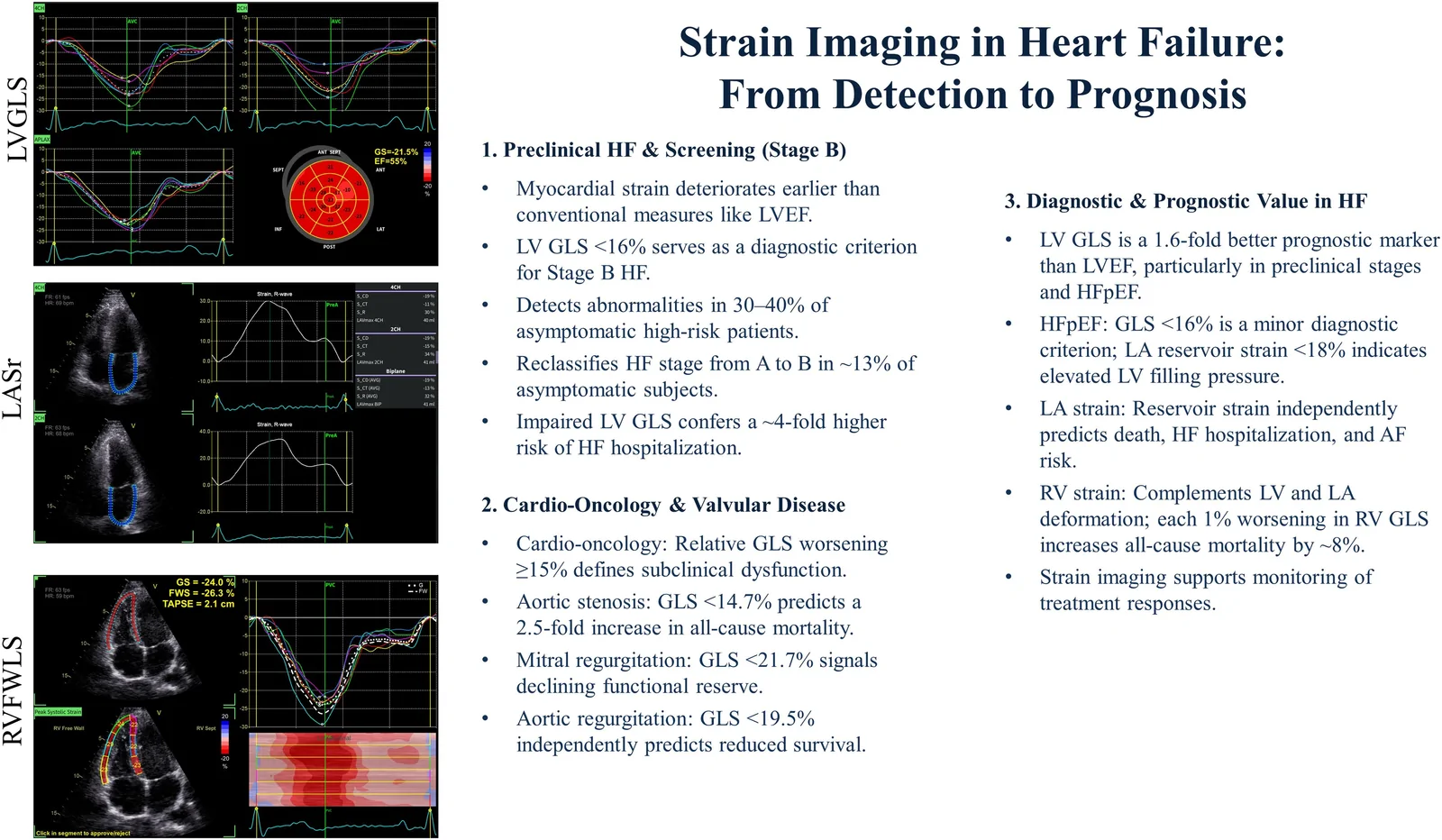

## Introduction

Accumulating evidence indicates that the traditional left ventricle ejection fraction (LVEF)-centric approach to cardiac functional assessment no longer meets the contemporary needs of clinical cardiology. LVEF, while historically central to HF classification, detects dysfunction relatively late in the disease process and provides limited insight into myocardial mechanics. Myocardial strain imaging by speckle-tracking echocardiography (STE)—encompassing left ventricle (LV), left atrial (LA), and right ventricle (RV) deformation—has therefore emerged as a modality that substantially expands the diagnostic and prognostic capabilities of conventional echocardiography.^1–4^

STE offers superior sensitivity for detecting early, subclinical myocardial dysfunction, provides incremental prognostic information, and enhances the monitoring of therapeutic responses and clinical decision-making.^3,5,6^ Due to its high feasibility, reproducibility, and technical advantages, such as angle independence, STE delivers deeper insight into myocardial mechanics beyond traditional volumetric indices. Importantly, strain parameters are less dependent on LV geometry and loading conditions than LVEF, thereby revealing diagnostic information frequently missed by conventional assessment.^7,8^

Strain imaging captures myocardial mechanics and provides diagnostic information across the entire HF spectrum, from early disease detection to advanced phenotyping. Among the three principal components of LV deformation—longitudinal, circumferential, and radial—longitudinal strain has the greatest clinical applicability. It reflects the function of subendocardial longitudinal fibres, which are particularly vulnerable to early injury and fibrotic remodelling in cardiovascular disease.^9–11^ Consequently, reduced longitudinal strain serves as a sensitive marker of early myocardial impairment, often preceding abnormalities in LVEF, which is governed predominantly by circumferential and radial mechanics^5,12–14^ . Because LV longitudinal deformation and LVEF interrogate different aspects of systolic function, they do not provide identical diagnostic or prognostic information.^1,7^

The LA strain has become an extensively used marker of diastolic function, integrating both LV and LA mechanics.^15,16^ LA reservoir and contractile strain correlate closely with LV filling pressures, and reservoir strain is now incorporated into formal diastolic guidelines.^17–20^ Beyond diastology, LA strain—due to its unique pathophysiological position—serves as a powerful predictor of heart failure and atrial fibrillation,^13,21–23^ reflecting the cumulative burden of elevated filling pressures and atrial remodelling.^24,25^

RV strain is a sensitive marker of early RV dysfunction and possesses strong prognostic value. It contributes to HF phenotyping and to the characterization of myocardial disease involvement, including conditions with RV pressure or volume overload and those primarily affecting RV myocardium. By detecting subtle RV impairment before overt functional decline, RV strain strengthens risk stratification and supports more comprehensive HF evaluation.^4,26–28^

Examples of LV, LA, and RV strain curves are shown in *Figures 1–3*. *Table 1* presents normative ranges of LV, LA, and RV deformation.

**Figure 1:**
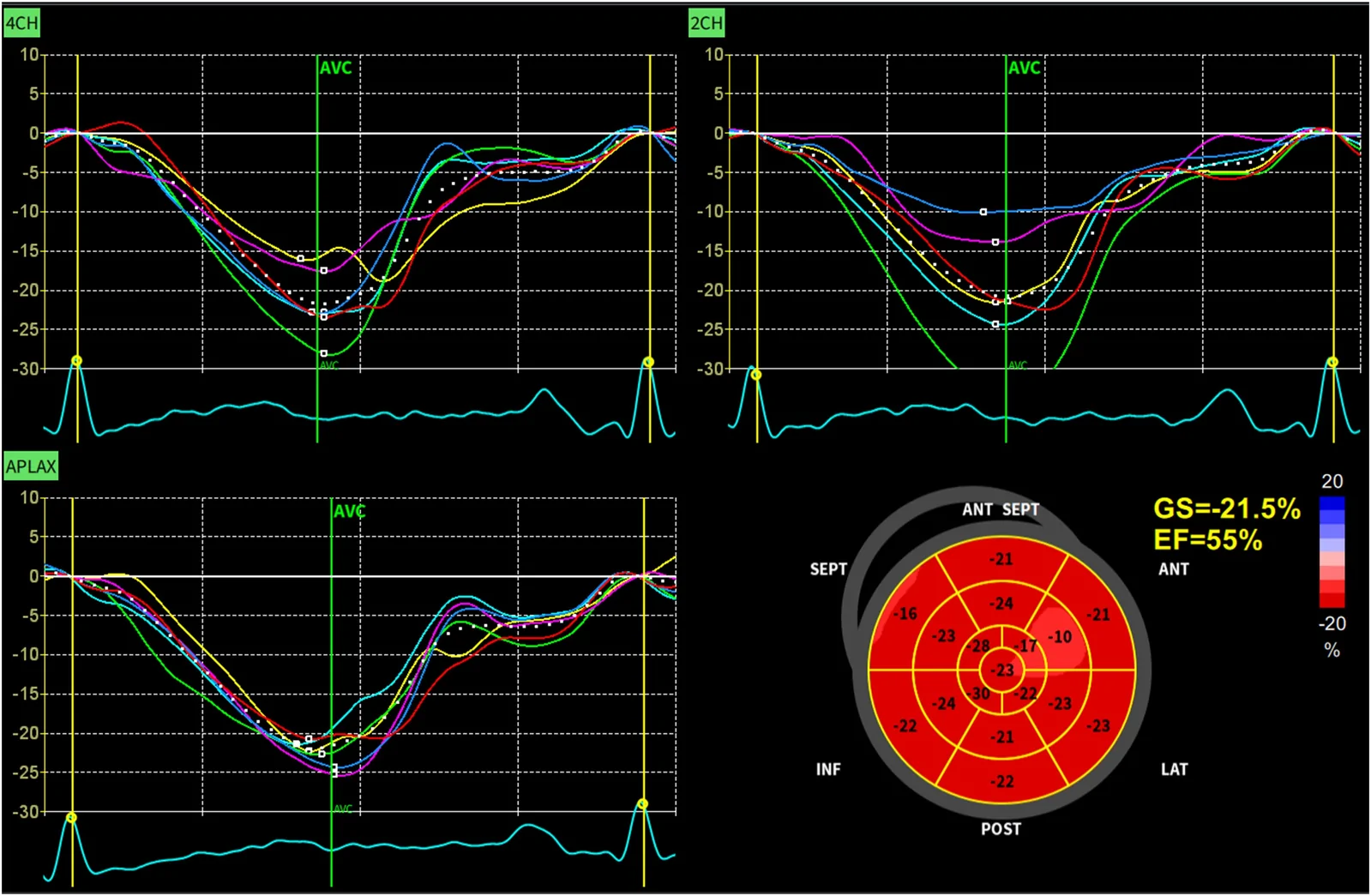
Representative curves and bull’s-eye map of left ventricular longitudinal strain

**Figure 2:**
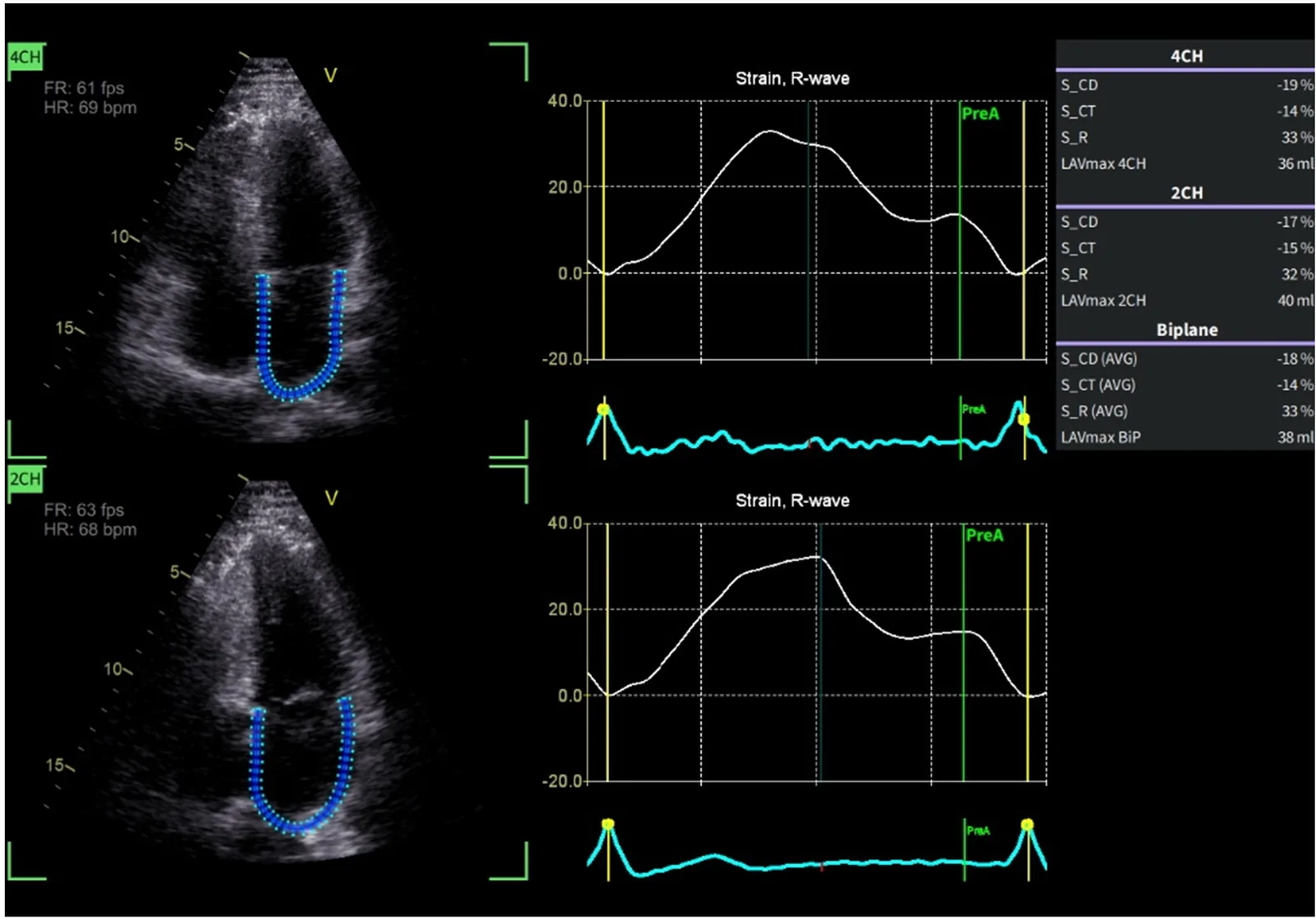
Representative curves of left atrial strain demonstrating reservoir, conduit, and contractile phases

**Figure 3:**
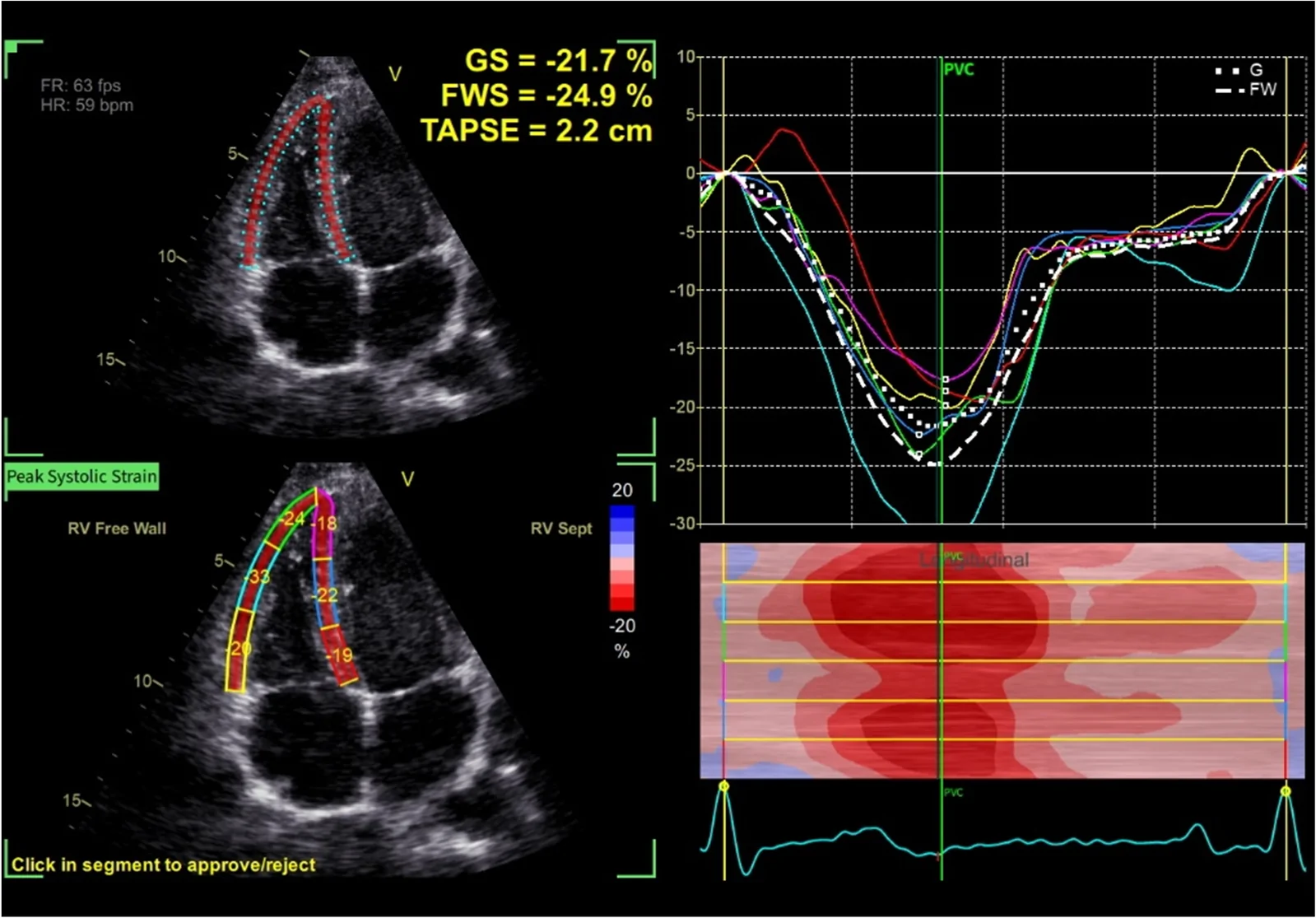
Representative curves of right ventricular longitudinal strain

**Table 1:** Normative values of myocardial strain according to Thomas ***et al.*^29^**

Chamber and parameter	Normal range	Borderline	Abnormal/pathological
Left ventricular global longitudinal strain	>18%	16%–18%	<16%
Left atrial reservoir strain	30%–60%	23%–30%	<23% (Elevated LV Filling Pressures: LASr <18%)
Right ventricular free wall longitudinal strain	Men: >20% Women: >21%		Men <20% Women <21%

LASr, left atrial reservoir strain.

## Preclinical heart failure

LV global longitudinal strain (GLS) plays a meaningful diagnostic and risk-stratifying role in preclinical HF. According to the scientific statements, its reduced values (<16%) are among the diagnostic criteria for stage B HF (SBHF).^30–33^ Abnormal LV GLS has been documented in 30%–40% of asymptomatic patients with HF risk factors, being the most common abnormality indicating SBHF (33% vs 31% for left atrial enlargement [LAE], 13% for left ventricular hypertrophy [LVH], and 12% for elevated E/e’).^34,35^

The addition of LV GLS to the diagnostic portfolio may reclassify the HF stage from A to B in 13% of patients.^36^ In subjects with metabolic disorders (diabetes mellitus, obesity, metabolic syndrome), GLS impairment is associated with insulin resistance, a control and adiposity metrics, such as body mass index (BMI), waist circumference (WC), and waist-hip ratio (WHR).^37–40^

LV GLS has proven to be a strong predictor of progression to symptomatic HF (stage C). Its values worse than 16%–18% in asymptomatic and community-based populations were associated with the risk of mortality and incident HF, providing incremental prognostic value over clinical factors and conventional echocardiographic indices, such as LVEF, left ventricular mass index (LVMI), left atrial volume index (LAVI), and E/e’.^35,41–43^

The identification of high-risk asymptomatic individuals may inform the need for early interventions aimed at preventing clinical deterioration. The prognostic relevance of LV GLS in preclinical HF is further underscored by the observation that impaired longitudinal deformation confers a four-fold higher risk of HF hospitalization compared with individuals exhibiting normal GLS.^42^ As demonstrated by AI-based phenotyping, the coexistence of reduced GLS and abnormal diastolic markers substantially heightens the risk of transition to symptomatic HF.^44^

## Surveillance in cardio-oncology

The LVEF-based monitoring of oncologic patients undergoing potentially cardiotoxic therapy is suboptimal, given the measurement variability and late appearance of these marker abnormalities.^12^ The pre-treatment GLS impairment below 15% was a better predictor of HF risk than LVEF <50%, and in contrast to LVEF, was independently associated with all-cause mortality in patients with leukaemia receiving anthracyclines.^5,45^ Current guidelines recommend the use of GLS to monitor cardiodepressive effects of oncotherapy, especially anthracyclines or human epidermal growth factor receptor 2-targeted treatments. A 15% relative worsening from baseline is considered subclinical LV dysfunction, which precedes LVEF decline and supports timely therapeutic adjustments, including pharmacological cardioprotection.^46–48^

## Valvular heart disease

Although LVEF remains a central metric in the management of valvular heart disease, assessment of GLS enables detection of subclinical LV dysfunction, supporting an earlier valve intervention in asymptomatic subjects.

Decreased GLS portends an adverse outcome in aortic stenosis (AS), irrespective of clinical symptoms. A meta-analysis of 10 studies encompassing asymptomatic individuals with preserved LVEF demonstrated that GLS <14.7% was associated with a 2.5-fold increase in all-cause mortality.^49,50^ Moreover, similarly decreased GLS to <15% predicted the presence of replacement fibrosis on cardiac magnetic resonance (CMR) in patients with moderate or severe AS.^51^ The prognostic value of impaired GLS in less advanced stages of AS was documented among patients with a moderate disease severity and preserved LVEF, where GLS <16% conferred a 2-fold higher risk of mortality—comparable to that in patients with LVEF <50%.^52^

In contrast to LV pressure overload in AS—where prognostic GLS thresholds fall within ranges considered abnormal in healthy individuals—volume-overload valvular disease (mitral regurgitation [MR] and aortic regurgitation [AR]) is marked by cutpoints that remain within the normative spectrum. Early in the natural history of MR and AR, increased LV myocardial stretch enhances systolic shortening via the Frank–Starling mechanism, resulting in high-normal or even supranormal GLS. As the disease progresses and the LV functional reserve declines, this compensatory response wanes, leading to falling GLS values. Thus, LV longitudinal deformation may still appear ‘normal,’ while values in the mid- to lower-normal ranges herald symptom progression rather than assure preserved functional status.

In asymptomatic AR without other echocardiographic indications for intervention, GLS <19.5% predicted reduced survival, with each 1% worsening associated with an 11% increase in mortality risk.^53^ Similarly, in asymptomatic severe primary MR with normal LVEF and LV size, GLS <21.7% identified a higher mortality risk, and every 1% deterioration translated into a 60% rise in death risk.^54^ The prognostic value of GLS was also demonstrated in patients with severe symptomatic MR scheduled for transcatheter edge-to-edge repair, in which GLS <14.5% independently predicted 1-year mortality.^55^

Despite emerging evidence, guideline-derived recommendations for the use of GLS in valvular heart disease remain very general, and LV longitudinal deformation is viewed only as a supportive measure in clinical decision-making.

**LA strain in asymptomatic heart failure stages.** The emergence of LA strain as an effective diagnostic marker lends promise for its implementation in preclinical HF. In a multicentre registry of 745 patients spanning stages 0/A–C, LA reservoir strain discriminated stage B from stage 0/A and stage C from 0/A.^56^ To date, further studies are needed to establish LA deformation as a factor identifying early asymptomatic cardiac impairment consistent with stage B HF.

## Diagnostic value of strain imaging across HF phenotypes

Abnormalities of myocardial deformation are common across HF categories and provide important diagnostic value.

**HFpEF.** Abnormal GLS values are observed in 52–65% of heart failure with preserved ejection fraction (HFpEF) patients enrolled in clinical studies^57–60^ and in up to 76% of patients who experienced acute HFpEF decompensation.^61^ Given this high prevalence, GLS <16% has been incorporated as a minor diagnostic criterion for HFpEF within the HFA–ESC diagnostic framework.^30^ This highlights the utility of GLS in distinguishing cardiac HFpEF non-cardiac causes of dyspnoea.

Beyond global longitudinal strain measurement, polar maps of segmental deformation can contribute to diagnostic pathways in specific HFpEF aetiologies. The ‘apical sparring’ pattern, indicating relative preservation of apical function, suggests cardiac amyloidosis (*Fig 4*), although a similar composition may occur in sarcomeric hypertrophic cardiomyopathy (HCM), AS, or chronic kidney disease, limiting specificity of this finding.^22,50,62–66^ In Anderson-Fabry disease, early involvement of the basal-to-mid infero-posterolateral segments can be detected as regional longitudinal strain reduction (*Fig 5*).^67^

**Figure 4:**
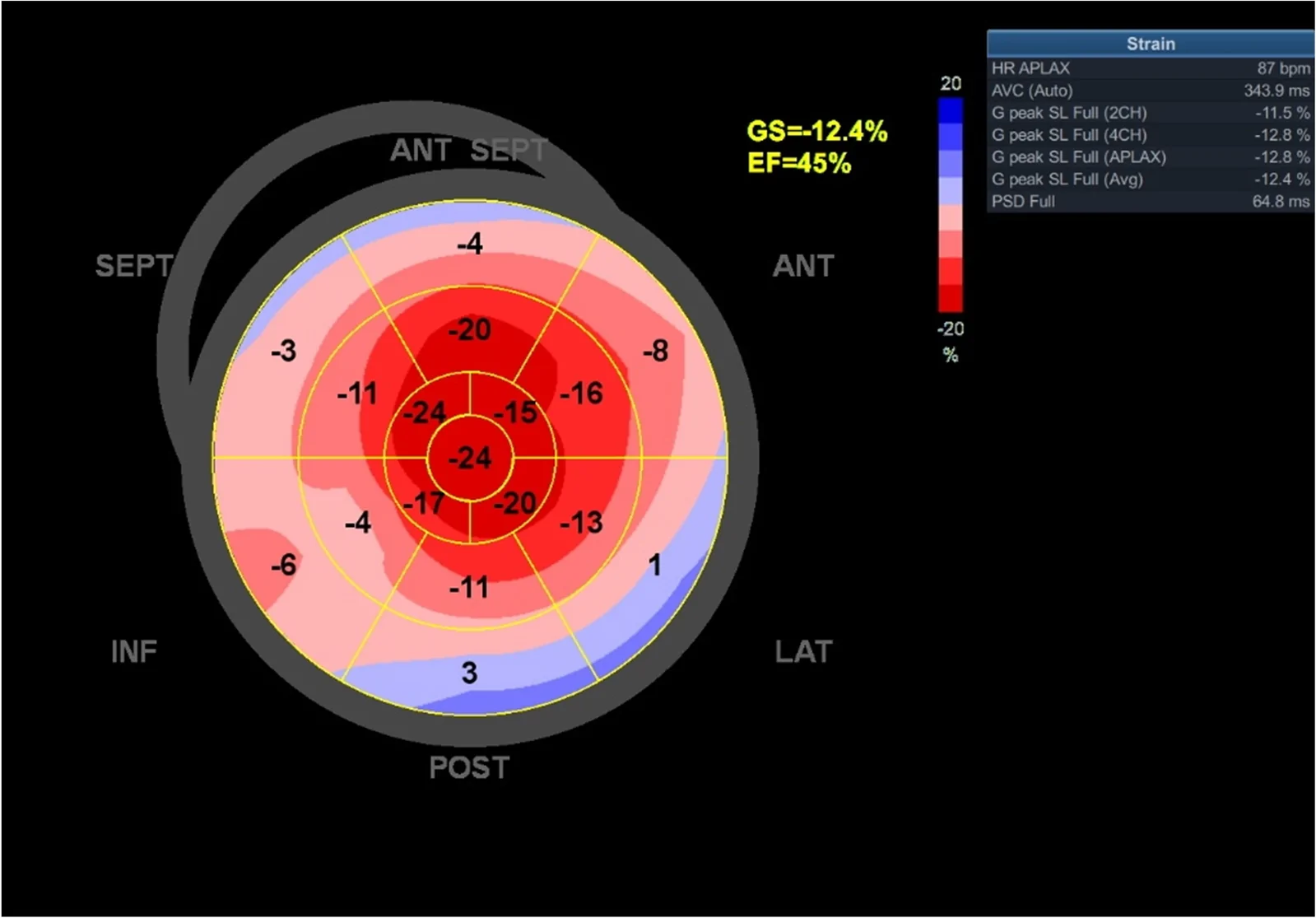
Longitudinal strain bull’s-eye map in cardiac amyloidosis showing the ‘apical sparing’ pattern, characterized by preserved strain in the apex and reduced strain in the basal and mid-ventricular segments

**Figure 5:**
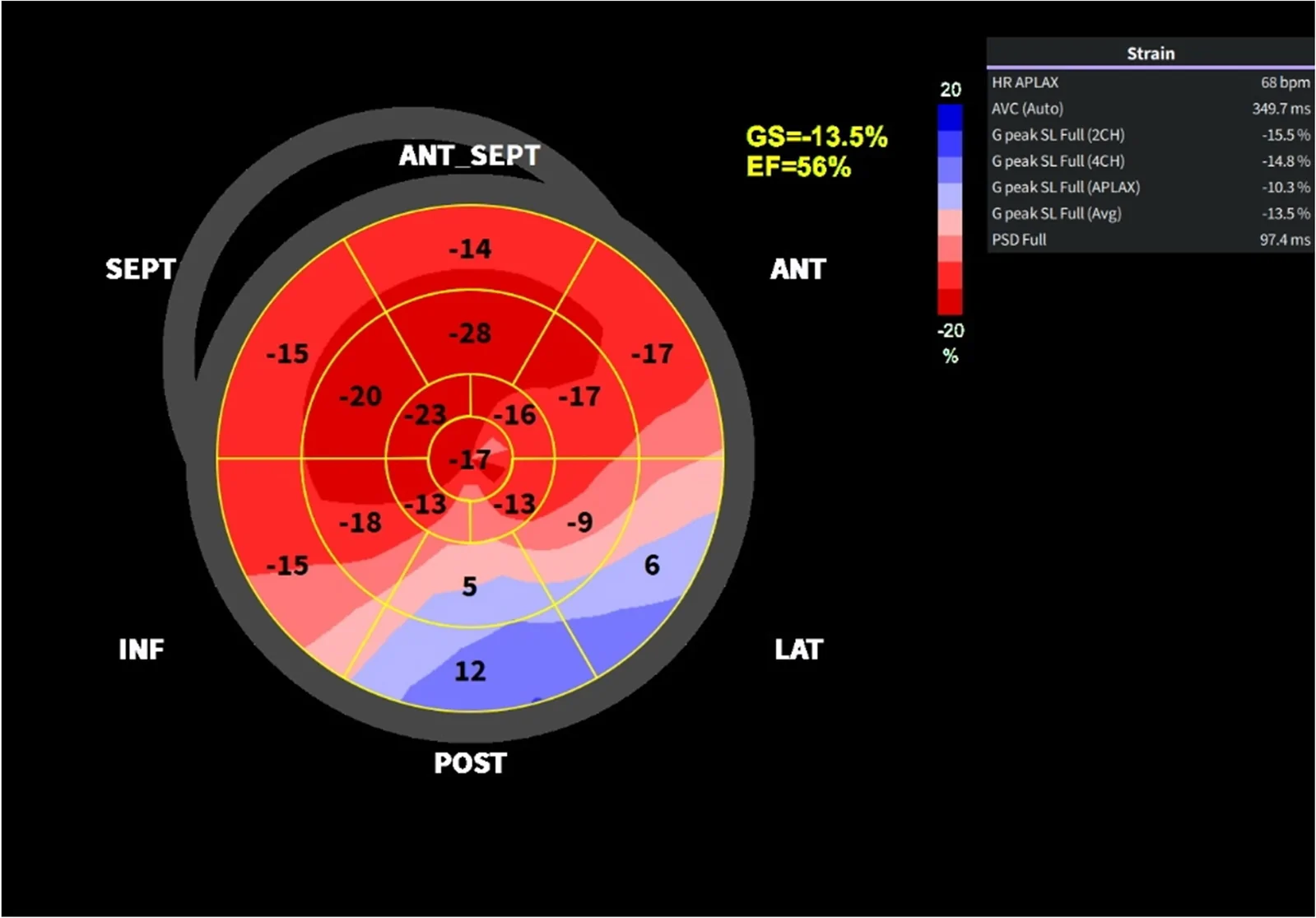
Longitudinal strain bull’s-eye map in Anderson-Fabry disease, demonstrating regional impairment primarily in the basal and mid-postero-lateral segments of the left ventricle

LA reservoir and contractile strains correlate linearly with LV filling pressure.^18,68^ LA reservoir strain is a better discriminator of LV diastolic dysfunction than LAVI or E/e’.^18,69–71^ It deteriorates earlier than LAVI in response to pathological conditions and ageing^70,72^ and shows clear associations with exercise capacity and LA collagen deposition.^20,73^ Accordingly, LA reservoir strain with a cutpoint of 18% has been included in algorithms for identifying and grading LV diastolic dysfunction.^19,74^

**HFmrEF.** The degree of GLS impairment may provide additional diagnostic information on disease trajectory in this heterogenous category. Mildly reduced or near-normal GLS values suggest HFpEF-like physiology with a tendency towards LVEF stability, whereas clearly reduced longitudinal deformation may indicate the risk of ongoing LVEF deterioration and a higher likelihood of transition to HFrEF.^6,16,75,76^

**HFrEF.** GLS is typically abnormal in parallel with reduced LVEF. Increased LV mechanical dispersion, defined as an SD of time-to-peak segmental longitudinal strain exceeding 60 ms, predicts ventricular arrhythmias and supports decisions regarding HF electrotherapy.^77,78^ The TARGET and STARTER trials demonstrated that using LV radial strain to identify the latest activated myocardial segment for CRT lead placement improved reverse remodelling, electromechanical synchrony, and clinical status.^79,80^

## Strain imaging for risk stratification in HF

GLS is a strong predictor of outcome across HF phenotypes and clinical presentations. Its predictive value remains independent of clinical staging and neurohormonal activation, despite the associations with these factors.^81^ A meta-analysis encompassing a broad spectrum of observational studies demonstrated that GLS is, on average, a 1.6-fold better prognostic marker in HF than LVEF, with particular utility in preclinical stages and HFpEF.^82^

GLS exhibits a more linear relationship with mortality in HF than LVEF. The distribution of deaths forms a single peak across GLS values of 6–12%, while LVEF shows a two-peak pattern that corresponds to the LVEF ranges typical of HFpEF and HFrEF.^83^

The prognostic signals of LVEF and GLS differ, and GLS offers incremental prognostic value that is independent of LVEF.

**HFpEF**. The prognostic utility of GLS in HFpEF has been underscored by multiple papers. Abnormal values <15.8–16% predicted both mortality and HF hospitalization., and each 1% decrease was associated with a 19% higher risk of death and an 8% increased risk of death or rehospitalization.^57,60,61^

Notably, HFpEF patients with reduced GLS had worse outcomes than their peers with normal longitudinal deformation, despite similarities in LVEF and diastolic status.^84^

**HFmrEF.** The prognostic role of GLS in HFmrEF is particularly important given the limited risk-stratification achievable with LVEF alone in this category. HFmrEF patients with GLS <10% have been shown to exhibit symptoms and outcomes comparable to those observed in HFrEF.^83^ Moreover, substantially reduced LV longitudinal deformation (<11%) has been associated with an increased risk of cardiovascular death and with subsequent decline in LVEF below 40% during follow-up.^75,85^

**HFrEF.** GLS retains strong prognostic value in HFrEF, providing more robust predictive information for mortality and hospitalization than LVEF. Reduced GLS, defined by cut-off values of 6.95%–10.3%, independently predicted clinical risk even after multivariable adjustment for demographic and clinical factors, ischaemic aetiology, natriuretic peptide levels, and diastolic abnormalities.^86–89^ The likelihood of death from all causes increased by 15% for every 1% absolute deterioration in GLS.^86^

**LA strain.** Existing data confirm the prognostic relevance of LA strain in HF. Meta-analytical papers including patients across all HF categories demonstrated that LA reservoir strain is an independent predictor of mortality and cardiac hospitalization, and that adding this parameter to risk-stratification models incorporating clinical factors, N-terminal pro-B-type natriuretic peptide (NT-proBNP), LAVI, LVEF, and LV GLS significantly improves their predictive performance.^16,90^ Each 1-unit reduction in LA reservoir strain was associated with a 5% increase in the combined endpoint of mortality and HF hospitalization, and the prognostic strength of LA strain did not differ between acute and chronic HF cohorts.^16^

More risk-stratifying data for LA strain come from HFpEF cohorts, where its associations with exercise capacity, LV filling, and pulmonary vascular resistance closely parallel its prognostic relationships.^73,91–94^ This pattern is consistent with HFpEF pathophysiology, in which LA dysfunction reflects the cumulative burden of impaired LV relaxation, elevated filling pressures, and increased pulmonary vascular load. However, in selected HFrEF populations, LA reservoir strain <10.8% predicted poor clinical outcomes and failure to recover LVEF above 40%.^95^

The prognostic role of LA deformation in HF also encompasses AF risk prediction. Both LA reservoir strain (cutpoint of 18%) and LA contractile strain were associated with incident AF in patients with HF in sinus rhythm.^94,96^

**Right atrial strain.** Analogous to LA strain, right atrial (RA) deformation reflects right-sided filling pressures and atrioventricular interactions, offering insight into adverse right-sided cardiac remodelling and the haemodynamic burden of systemic venous congestion. In HF, impaired RA strain has been demonstrated as an independent predictor of unfavourable outcomes, including mortality and HF hospitalization.^97–100^ The presence of biatrial dysfunction, evidenced by concomitantly reduced LA and RA strain, confers particularly elevated clinical risk,^101,102^ highlighting the integrative role of atrial mechanics in HF pathophysiology.

The prognostic relevance of RA strain has also been shown in cardiac amyloidosis, where reduced RA deformation was significantly associated with poor outcomes even after adjustment for LV GLS, RV GLS, and LA strain.^66^ These findings underscore that RA strain captures unique aspects of right-sided myocardial involvement not fully reflected by other chamber-specific strain parameters.

However, it should be emphasized that the implementation of RA strain into routine clinical practice remains tempered by the lack of standardized reference values and substantial measurement variability, particularly in conditions with marked right-sided pressure overload.^103^

**RV strain**. RV longitudinal deformation has emerged as a valuable predictor of adverse outcomes in HF, demonstrating superior prognostic accuracy over conventional markers such as tricuspid annular plane systolic excursion (TAPSE) or fractional area change (FAC).^104^ A meta-analysis of 24 studies including 8738 patients across all HF categories showed that worsening RV GLS or RV free-wall longitudinal strain (RV FWLS) independently predicted mortality and the composite of mortality/HF hospitalization, with consistent associations in the subgroup with LVEF <45%. Each 1% worsening in RVGLS and RV FWLS was independently associated with an 8% and 5% increased risk of all-cause mortality and a 10% and 6% higher risk of the composite outcome, respectively.^105^ These quantitative relationships highlight the steep prognostic gradient captured by RV deformation. Individual studies demonstrated significant risk-stratifying value of RV deformation in stable HFrEF (prognostic cutpoint for RV GLS 19%), acute and end-stage HFrEF,^106–108^ as well as in HFpEF (prognostic cutpoint for RV GLS 17.5%).^109,110^

RV strain also contributes to diagnosis, monitoring of disease progression, and prognostic assessment in arrhythmogenic right ventricular cardiomyopathy, where RV FWLS <20% has been linked to adverse outcomes.^111,112^

Taken together, RV strain provides powerful prognostic information that complements LV and atrial deformation indices, enabling more refined risk stratification in HF. Its ability to detect subtle RV dysfunction—often before conventional parameters deteriorate—positions RV strain as an essential component of contemporary HF evaluation.

## Strain imaging in monitoring responses to heart failure therapies

Strain imaging is increasingly used to guide therapeutic decision-making and to monitor treatment response across the HF spectrum. Because myocardial deformation parameters respond earlier than conventional measures, such as LVEF or natriuretic peptides, they offer a sensitive window into myocardial and haemodynamic improvement. Early changes in strain frequently precede volumetric reverse remodelling, making strain-guided monitoring particularly valuable during the initial phases of therapy optimization.^26^

In patients receiving angiotensin receptor-neprilysin inhibitors (ARNI) therapy, studies in HFrEF have shown that early improvements in LV GLS within the first 3 months predict long-term LV recovery, with >15% increases in LVEF observed at 12-month follow-up among those who demonstrate early strain response.^113^ Larger cohort analyses of sacubitril/valsartan therapy^114^ have further demonstrated that baseline LVGLS is independently associated with subsequent LV reverse remodelling, underscoring the role of strain as a sensitive marker of treatment responsiveness.

Early improvement in LVGLS within 3 months—sustained at 12 months—has also been documented in HFrEF patients treated with combined ARNI and sodium/glucose cotransporter 2 (SGLT2) inhibitors, indicating that myocardial deformation imaging can detect early recovery and track therapeutic effects across multiple pharmacologic classes.^115^ Extending this evidence base, the randomized DAPA-ECHO trial (Effects of Dapagliflozin on cardiac deformation and clinical outcomes in heart failure with reduced and mildly reduced ejection fraction) showed that adding dapagliflozin to otherwise optimal medical therapy (except SGLT2 inhibitors) in non-diabetic HFrEF and HFmrEF patients resulted in significant improvements in LV GLS, RV GLS, and LA strain at 6-month follow-up.^116^ These chamber-specific improvements highlight the ability of strain imaging to capture multidimensional cardiac recovery beyond LV systolic function alone.

Collectively, these studies indicate that strain parameters—particularly LVGLS—provide early, actionable predictive value for therapeutic responses in HF. Strain-guided monitoring may therefore complement clinical assessment and biomarkers, enabling more precise and timely evaluation of treatment efficacy.

## Methodological considerations

Two-dimensional (2D) STE remains the core modality for strain assessment in HF due to its feasibility, reproducibility, and extensive validation. Alternative approaches exist but have more limited clinical applicability.^117,118^

Three-dimensional STE offers the theoretical benefit of capturing all directional components of deformation within a single-beat acquisition. However, its lower spatial and temporal resolution reduces tracking reliability and measurement accuracy, thereby limiting broad clinical adoption.

CMR-based strain techniques are independent of acoustic windows and demonstrate generally acceptable agreement with STE, particularly for longitudinal and circumferential deformation. Despite these advantages, high cost, limited availability, longer examination times, and patient-related constraints (claustrophobia, device implantation) restrict the widespread use of CMR for strain assessment in HF.^1,119^

Automated strain analysis available on commercial platforms facilitates integration of deformation imaging into routine diagnostic workflows.^120^ Continued development of AI-enhanced algorithms may further improve precision, reduce operator dependence, and support real-time clinical decision-making, which is particularly relevant in HF, where serial strain assessment can guide therapy optimization and risk stratification.

Strain measurements rely on post-processing algorithms and vendor-specific implementations, making standardization more challenging than for volumetric indices such as LVEF. This is less problematic for LV deformation, where substantial progress has been made towards harmonizing inter-vendor differences, and the lower limit of normality for GLS (∼16%) is now largely independent of the software used.^121^

**Myocardial work**, derived from combining LV pressure estimates with strain curves, represents an emerging complementary tool. By incorporating afterload, myocardial work provides a more physiologically grounded assessment of LV performance and may enhance risk stratification and therapy monitoring in HF beyond strain alone, but more evidence is needed before it can be regarded as a viable option.

## Conclusions

Strain imaging has expanded from a specialized technique to a fundamental part of contemporary HF evaluation. By capturing myocardial mechanics, not volumetric changes alone, strain facilitates earlier detection of dysfunction, more refined phenotyping across HF categories, and stronger prognostic stratification than conventional measures. It may also offer value for therapy monitoring, where early improvements in deformation parameters can identify responders and guide treatment optimization.

The future role of strain imaging will likely be determined by further standardization and wider adoption of automated and artificial intelligence (AI)-supported analysis. As HF management continues to shift towards personalized treatment strategies, deformation imaging is anticipated to serve as a pivotal component of integrated diagnostic protocols, enhancing diagnostic accuracy, risk prediction, and therapeutic decision-making.

## Contributions

W.K. was responsible for the conceptualization of the review, drafting the manuscript, and critically revising it for important intellectual content. A.W. contributed to the development and refinement of the final text. Both authors participated in the literature search and read and approved the final manuscript for publication.

## Declarations

### Data Availability

No data were generated or analysed for this manuscript.
